# Changes in the Size of a Ruptured Pheochromocytoma after Transcatheter Arterial Embolization

**DOI:** 10.1155/2021/5568978

**Published:** 2021-04-04

**Authors:** Takahiro Ichikawa, Chikako Oyabu, Megumi Minamida, Yusuke Ichijo, Yoshitaka Hashimoto, Mai Asano, Hiroya Iwase, Toru Tanaka, Michiaki Fukui

**Affiliations:** ^1^Department of Endocrinology and Metabolism, Kyoto First Red Cross Hospital, Kyoto, Japan; ^2^Department of Radiology, Kyoto First Red Cross Hospital, Kyoto, Japan; ^3^Department of Endocrinology and Metabolism, Kyoto Prefectural University of Medicine, Graduate School of Medical Science, Kyoto, Japan

## Abstract

The spontaneous rupture of a pheochromocytoma is rare and can be potentially fatal. We report a case of a tumor size reduction of a ruptured pheochromocytoma after transcatheter arterial embolization (TAE). A 60-year-old Japanese woman was referred to the emergency department of another hospital with a sudden onset of left lateral pain. Computed tomography of the abdomen revealed adrenal hemorrhage with a 5.7 cm adrenal mass, and she was transferred to our hospital for treatment. Considering that she had marked hypertension (193/115 mmHg), we made a provisional diagnosis of left lateral pain due to a ruptured pheochromocytoma. She underwent TAE, and the hemorrhage was successfully controlled. She was started on oral doxazosin for hypertension. The dose of doxazosin was increased to the extent that orthostatic hypotension did not develop, and blood pressure was well controlled. After discharge, the tumor size gradually decreased to approximately 1.0 cm within six months. Six months after TAE, elective laparoscopic surgery was performed, and the diagnosis was confirmed by histopathology. We observed a decrease in the size of the ruptured pheochromocytoma after TAE. To reduce the risk of laparoscopic adrenal surgery, it may be useful to monitor the size of a ruptured pheochromocytoma after TAE before deciding the surgery time.

## 1. Introduction

Pheochromocytoma is a relatively rare catecholamine-producing neuroendocrine tumor that presents with various clinical symptoms such as hypertension, headache, palpitations, and sweating. Pheochromocytoma is reported to account for 0.2% of all hypertension cases [1]. The spontaneous rupture of adrenal tumors is a very rare condition; however, pheochromocytoma is the most common tumor that develops spontaneous adrenal hemorrhage with associated masses [2]. In the case of a ruptured pheochromocytoma, emergent surgery is associated with a high mortality rate; however, previously reported cases have shown that elective surgery after appropriate management may lead to good outcomes [3]. In the current study, we report an extremely rare case of a spontaneous rupture of an adrenal pheochromocytoma in which transcatheter arterial embolization (TAE) was applied for hemostasis, and elective laparoscopic resection was performed after tumor shrinkage.

## 2. Case Presentation

**Figure 1:**
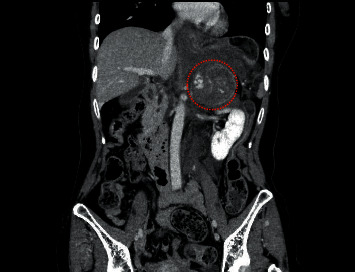
Computed tomography findings on admission. Contrast-enhanced coronal abdominal computed tomography showed a left 5.7 cm adrenal mass (red dots) and a large retroperitoneal hemorrhage.

A 60-year-old Japanese woman with no medical history was admitted to the emergency department of another hospital with a chief complaint of severe left lateral pain. She had no family history of endocrine disease including pheochromocytoma. Abdominal computed tomography revealed a left 5.7 cm adrenal mass with a large retroperitoneal hemorrhage (Figure 1), and the patient was transferred to our hospital for multidisciplinary treatment.

She was 149 cm tall and weighed 49 kg. On presentation, her vital signs and physical findings were as follows: body temperature of 37.3°C, pulse rate of 106 bpm, blood pressure of 193/115 mmHg, respiratory rate of 18/min, and Glasgow Coma Scale score of 15. These findings strongly suggested that her pain was caused by a ruptured pheochromocytoma. Laboratory tests revealed that her white blood count was 28180/*μ*L, hemoglobin was 11.2 g/dL, and hepatic enzymes were normal. However, her creatinine (Cre) and estimated glomerular filtration rate (eGFR) were 1.03 mg/dL and 43 ml/min/1.73 m^2^, respectively, which indicated decreased renal function. After arriving at our hospital, her blood pressure suddenly dropped to 62/44 mmHg. Ongoing hemorrhage was suspected because of sudden hypotension. Under a presumptive diagnosis of active bleeding from a ruptured left adrenal tumor suspected of pheochromocytoma, we decided to perform TAE to prevent the further deterioration of hemodynamics. Angiography was performed using a right femoral approach and showed that the tumor blood flow was supplied from a branch of the left adrenal artery. Thereafter, TAE was selectively performed for the left inferior adrenal artery by using gelatin sponge particles and coiling. No further tumor vascularity was demonstrated by subsequent contrast injection. Her hemodynamics stabilized after TAE, and systolic blood pressure ranged from 150–170 mmHg. After admission, she was treated with intravenous nicardipine and oral doxazosin. Intravenous phentolamine was not administered to this patient because phentolamine was not available in our hospital.

On the second day of admission, hormonal assays were performed and showed markedly elevated levels of plasma metanephrine at 952 pg/mL (reference range <130 pg/mL), plasma normetanephrine at 3150 pg/mL (reference range <506 pg/mL), urinary metanephrine at 6.64 *μ*g/mg Cr (reference range <0.2 *μ*g/mg Cr), and urinary normetanephrine at 7.22 *μ*g/mg Cr (reference range <0.3 *μ*g/mg Cr). On the third day of admission, abdominal computed tomography was performed and showed a significant resolution of the retroperitoneal hematoma. She was discharged on day 11 after TAE without any symptoms. At discharge, the systolic blood pressure was improved to 130 mmHg or less by oral doxazosin 4 mg/day alone. On the day after discharge, an iodine-131 metaiodobenzylguanidine (MIBG) scan was performed, and results showed only a significant left adrenal uptake.

**Figure 2:**
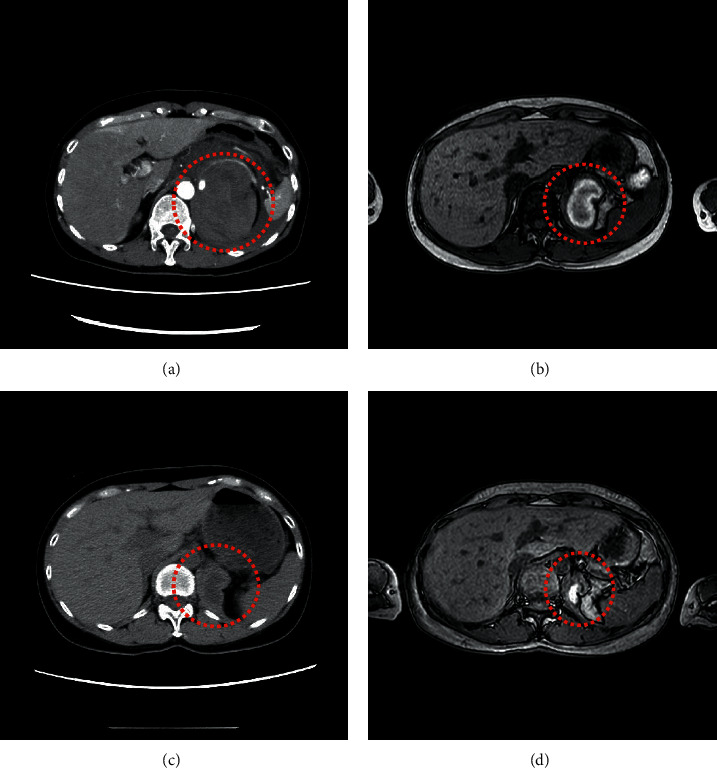
Change in imaging findings. (a) Contrast-enhanced axial computed tomography on admission indicated a left adrenal mass and a large retroperitoneal hemorrhage (red dots). (b) T1-weighted magnetic resonance imaging after a month discharge showed tumor shrinkage of a left adrenal mass and the reduction of hematoma (red dots). (c) Plain axial computed tomography three months after discharge indicated a 2.8 cm left adrenal mass and hematoma (red dots). (d) T1-weighted magnetic resonance imaging five months after discharge showed even more tumor shrinkage and the reduction of hematoma (red dots).

**Figure 3:**
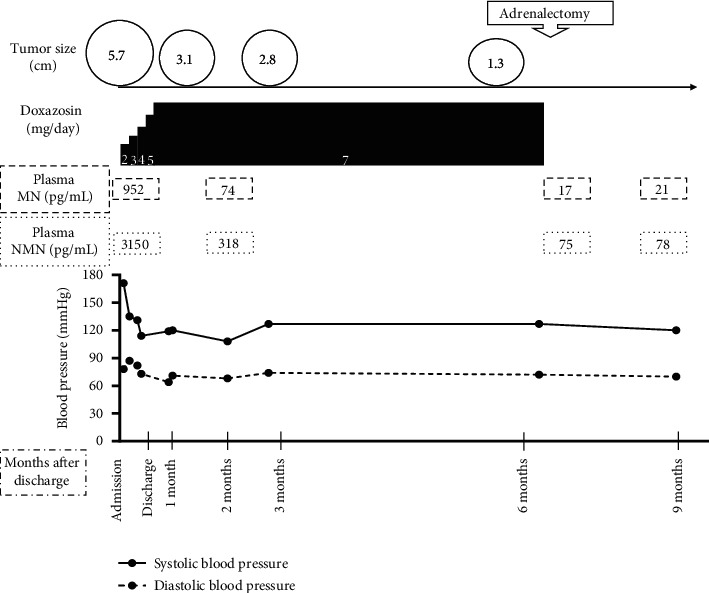
Time course of clinical data. Blood pressure was well controlled with a significant decrease in plasma levels of catecholamines as the tumor size decreased after TAE. MN, levels of plasma metanephrine; NMN, levels of plasma normetanephrine.

**Figure 4:**
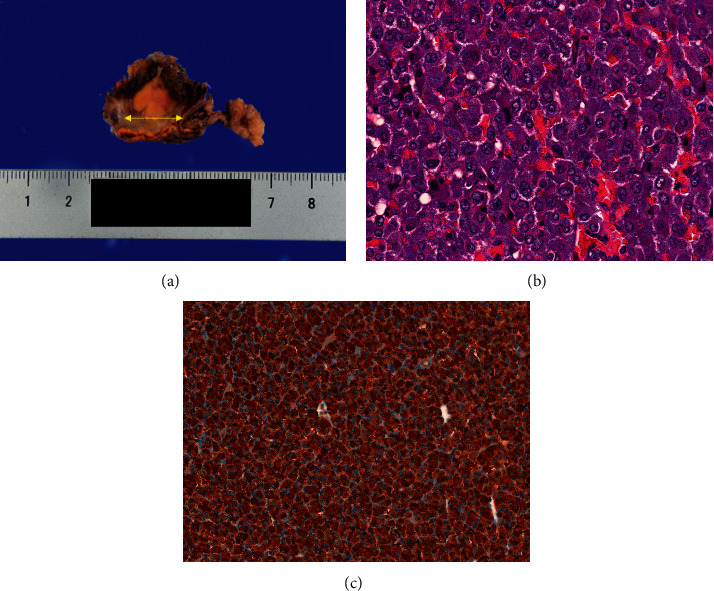
Histopathological findings of the resected adrenal gland. (a) The cut surface of the tumor. The left adrenal tumor (a double-headed yellow arrow) measured 13 mm × 12 mm × 11 mm. (b) The section showed the large and irregular cell nest arrangement of tumor cells (hematoxylin and eosin × 400). (c) The brown staining showed the expression of chromogranin A in the tumor × 200.

She was followed up at an outpatient clinic, and computed tomography or magnetic resonance imaging confirmed that the tumor size had spontaneously and gradually decreased. The tumor sizes were 3.1, 2.8, and 1.3 cm one month, three months, and five months after discharge, respectively (Figure 2). Tumor shrinkage prompted us to resect the tumor laparoscopically (Figure 3). Six months after TAE, laparoscopic left adrenalectomy was successfully performed with proper preoperative treatment by 7 mg/day of doxazosin. Pathological findings confirmed the diagnosis of an adrenal pheochromocytoma (Figure 4). The gross appearance of the resected tumor was 13 mm × 12 mm × 11 mm in size and was yellow and tan in color. Pathological examination revealed coagulative necrosis in the tumor, and the cytoplasm of the tumor cells was immunohistochemically positive for chromogranin A, synaptophysin, and S100. The Ki67 labeling index was <1%. A score of five was obtained from the grading system for adrenal pheochromocytoma and paraganglioma (histological pattern; large and irregular cell nest, one point; cellularity, high, two points; vascular or capsular invasion; presence, one point; and catecholamine type and noradrenaline type, one point) [4]. She was graded as moderately differentiated.

Doxazosin was stopped after surgical resection, and her blood pressure was stable. Plasma metanephrine and normetanephrine levels decreased to 21 and 78 ng/L, respectively, at three months postoperatively. Urinary metanephrine and normetanephrine levels also showed a significant decrease. At the half-year follow-up, her postoperative course was uneventful without any recurrence of pheochromocytoma, and levels of plasma metanephrine and normetanephrine remained within normal ranges.

## 3. Discussion

In this case report, we described an extremely rare case of a spontaneous rupture of an adrenal pheochromocytoma in which TAE was applied for hemostasis, and elective laparoscopic resection was performed after tumor shrinkage.

Pheochromocytoma is a rare catecholamine-secreting tumor of chromaffin cells. The major classic symptoms of pheochromocytoma are headache, palpitation, and diaphoresis with severe hypertension. However, some patients with pheochromocytoma are asymptomatic [5]; in such cases, tumor rupture may be the initial manifestations of pheochromocytoma. Tumor rupture is an extremely rare complication of pheochromocytoma; however, it affects the circulation system profoundly and can be potentially lethal. The mortality rate of a ruptured pheochromocytoma is reported to be 34% in the literature [3]. Although the mechanism of rupture is unclear, high intracapsular pressure may cause the necrosis of a pheochromocytoma [6]. Elective surgery for a ruptured pheochromocytoma following pharmacological therapy has a low mortality rate. However, emergent surgery for a ruptured pheochromocytoma is associated with a high mortality rate [3]. TAE has recently been reported as a suitable option for a ruptured pheochromocytoma to achieve hemodynamic stabilization and permit elective surgery [7]. We successfully controlled the hemorrhage due to a ruptured pheochromocytoma after TAE. However, among the past cases of a ruptured pheochromocytoma, one case resulted in death even after TAE [8]. In that case, TAE was performed 12 hours after the suspected of a ruptured pheochromocytoma, following the gradual decrease in blood pressure. Therefore, it is presumed that early treatment decision is required when diagnosing a ruptured pheochromocytoma.

In our case, the tumor size decreased after TAE. Upon admission to the hospital, the tumor size was 5.7 cm; however, consecutive radiological examinations demonstrated marked tumor shrinkage, and pathological examinations revealed a significant decrease to approximately 1.0 cm. This is the first report to demonstrate tumor shrinkage after TAE for a ruptured pheochromocytoma. The exact mechanisms of tumor shrinkage remain to be elucidated; however, starvation of blood supply by TAE and/or progressive necrosis due to spontaneous rupture may become a critical factor for the tumor shrinkage.

In this case, the preoperative large size of pheochromocytoma was considered to be a risk factor for intra- and postoperative complications. Bai et al.[9] showed that the radiographic large size of pheochromocytoma was an independent risk factor for postoperative cardiovascular morbidity and a cut-off value of the tumor size was 6.05 cm. Kwon et al.[10] also reported that the large size of pheochromocytoma was a risk factor for intraoperative hypertensive attack and a cut-off value of the tumor size was 4.25 cm and Liu et al. [11] showed that pheochromocytoma with tumor diameter greater than 5.00 cm was at risk of intraoperative massive blood loss. In this case, we worked closely with urologists to determine the surgery after confirming the sufficient shrinkage of the tumor and hematoma and the decrease in catecholamine levels. As a result, laparoscopic adrenalectomy was safely performed after tumor shrinkage. Elective surgery could be performed three months after discharge, based on the tumor size, the levels of catecholamines, and blood pressure; however, the corona virus 2019 pandemic forced to perform elective surgery at our hospital later than originally planned.

In our case, pathological findings suggested an intermediate risk of tumor metastasis but displayed no signs of recurrence during more than half a year of the postoperative follow-up period. Given that a recurrence of pheochromocytoma may occur even decades after surgery [12], further follow-ups will be mandatory for a long time in our case.

We reported a rare case in which significant tumor shrinkage was demonstrated after TAE for a ruptured pheochromocytoma. The tumor size monitoring may contribute to be a crucial determinant of timing of elective surgery in pheochromocytoma.

## Data Availability

The data used to support the findings of this study are available from the corresponding author upon request.
